# Respiratory Proteomics Today: Are Technological Advances for the Identification of Biomarker Signatures Catching up with Their Promise? A Critical Review of the Literature in the Decade 2004–2013

**DOI:** 10.3390/proteomes2010018

**Published:** 2014-01-22

**Authors:** Simona Viglio, Jan Stolk, Paolo Iadarola, Serena Giuliano, Maurizio Luisetti, Roberta Salvini, Marco Fumagalli, Anna Bardoni

**Affiliations:** 1 Department of Molecular Medicine, Biochemistry Unit, University of Pavia, Via Taramelli 3/B, Pavia 27100, Italy; E-Mails: simona.viglio@unipv.it (S.V.); serena.giuliano@unipv.it (S.G.); roberta.salvini@unipv.it (R.S.); abardoni@unipv.it (A.B.); 2 Department of Pulmonology, Leiden University Medical Center, Leiden 2333, The Netherlands; E-Mail: J.Stolk@lumc.nl; 3 Department of Biology and Biotechnologies, Biochemistry Unit, University of Pavia, Via Taramelli 3/B, Pavia 27100, Italy; E-Mail: fummar07@unipv.it; 4 Faculty of Science “Parc Valrose”, University of Nice “Sophia Antipolis”, FRE 3472 CNRS, LP2M Nice, France; 5 Department of Molecular Medicine, Division of Pneumology, University of Pavia & IRCCS Policlinico San Matteo, Via Taramelli 5, Pavia 27100, Italy; E-Mail: m.luisetti@smatteo.pv.it

**Keywords:** respiratory diseases, nano-LC-MS/MS, proteomics

## Abstract

To improve the knowledge on a variety of severe disorders, research has moved from the analysis of individual proteins to the investigation of all proteins expressed by a tissue/organism. This global proteomic approach could prove very useful: (i) for investigating the biochemical pathways involved in disease; (ii) for generating hypotheses; or (iii) as a tool for the identification of proteins differentially expressed in response to the disease state. Proteomics has not been used yet in the field of respiratory research as extensively as in other fields, only a few reproducible and clinically applicable molecular markers, which can assist in diagnosis, having been currently identified. The continuous advances in both instrumentation and methodology, which enable sensitive and quantitative proteomic analyses in much smaller amounts of biological material than before, will hopefully promote the identification of new candidate biomarkers in this area. The aim of this report is to critically review the application over the decade 2004–2013 of very sophisticated technologies to the study of respiratory disorders. The observed changes in protein expression profiles from tissues/fluids of patients affected by pulmonary disorders opens the route for the identification of novel pathological mediators of these disorders.

## 1. Introduction

Two decades ago, we had no idea whether methods that enabled scientists to move from the punctual analysis of individual proteins to the global investigation of all proteins expressed by a tissue/organism existed at all. A sort of revolution driven by technological advancements occurred in these years determined a change of the research strategy in the biochemical field and allowed proteomics to progress and become one of the most captivating branches of biochemistry. With the improvement in the sensitivity and specificity of analytical methods, a significant step forward has been achieved for the detection/quantification of all proteins expressed by an organism, even in extremely complex biological matrices or in the presence of very small sample amounts. Nevertheless, the potential of proteomics is still largely untapped, and it remains a speculation whether the application of these procedures resulted in profound implications for the study of human diseases. The number of publications in this field, which is almost decupling every year, may be an indirect answer to this question. Through the monitoring of protein expression patterns in clinical specimens, to date, these sophisticated technologies offer great opportunities to identify proteins that may be significantly associated with a specific disease status [1,2,3,4,5,6,7,8]. Obviously, well-standardized protocols are proposed not only as a way of searching for putative biomarkers of diseases, but also to provide a wealth of additional information on: (i) protein-protein interactions; (ii) the extent of post-translational modifications that significantly contribute to protein function; and (iii) the precise quantity of a protein in the sample considered. In other words, the ability of proteomics to provide insight into both specific and system-level changes in cell, tissue and human physiology has accelerated progress in the elucidation of a variety of multi-factorial pathological conditions, including less commonly diagnosed disorders [9,10,11,12,13].

Obviously, the establishment of a simple, robust and high throughput protein profiling system is extremely important from the point of view of clinical proteomics. Such a system would in fact help a large number of human tissues/biological fluids to be quantitatively analyzed for expressed proteins in a routine and reproducible manner. The most widely used proteomic tools so far developed are two-dimensional gel electrophoresis-mass spectrometry (2DE-MS), liquid chromatography-mass spectrometry (LC-MS) and capillary electrophoresis-mass spectrometry (CE-MS). Despite their excellent resolving power, gel-based approaches may have serious limitations associated with the difficulties in reproducibility and in protein identification, in particular if low-abundant proteins are concerned. These limitations not taken into account, 2DE/MS remains the “conventional” procedure applied to the differential (control *vs.* diseased case) analysis of biological samples [14,15,16,17,18,19,20]. On the other hand, the fact that many of these draw-backs are overcome by LC-MS and CE-MS indicates the gel-free methods as the ideal approaches for large-scale protein measurement across multiple biological samples. Naturally, the more advanced the technology applied is, the more likely that thousands of proteins may be identified and quantified, even in minute amounts of biofluids. Based on this hypothesis, the rapid advance of technology, along with a revolution in bioinformatic methods for analyzing multi-dimensional data have recently brought the coupling of evolved capillary-based LC separations online with tandem mass spectrometry (MS/MS). The current report was designed to critically review the results produced, over the past ten years, by the combination of micro- and nano-LC/CE with MS/MS in the area of respiratory diseases. Despite that the proteomics in this field has not been used yet as extensively as in other fields, several applications of micro/nano-procedures for different lung fluids and tissues have ultimately reached a depth of analysis never observed before. This has allowed for the generation of protein profiles that are useful for exploring protein-based pathological mechanisms and/or discovering new potential therapeutic targets for a variety of pulmonary disorders, including lung cancer, asthma, chronic obstructive pulmonary disease (COPD) and cystic fibrosis (CF).

The present status of lung proteome, with specific examples from pulmonary studies that have evaluated cell lysates, serum/plasma, bronchial/lung biopsies, bronchoalveolar lavage, exhaled breath condensate, sputum and urine, will be discussed below. 

## 2. Outline of the Article

At least four excellent reviews in the field of pulmonary proteomics have been published to date [21,22,23,24]. While some of these articles were focused on highly-specific topics, including CF, COPD and allergy/asthma [21,22,23], that of Hirsch *et al.* [24] was indeed very comprehensive and provided general information on the status of the most promising proteomic methods and some of their applications to pulmonary research until 2004. The conclusion of this report was that “*The ongoing rapid evolution in separation science, mass spectrometry and bioinformatics will continue to stimulate the investigation of lung proteome and lead to new insights in the near future.*” After a decade of “furious activity”, the proteomics of pulmonary diseases seems to be catching up with its promise, a good number of publications supporting the view of proteomics as a decisive methodological tool for the identification/characterization of disease-associated proteins.

This report further expands on the prior work conducted by Hirsch and colleagues, this being its ideal prosecution, since it covers the literature from 2004 until the present. It illustrates the results obtained from the application of the most advanced proteomic procedures (micro/nano-LC-MS/MS in particular) on different categories of biological samples from a variety of acute and chronic lung disorders.

Seven out of eleven chapters of this article will address the proteomic data relative to a specific lung disease. Since different fluids/tissues may serve as a rich source of information for the same disorder, specific paragraphs inside each chapter will be dedicated to the presentation of the proteomic profile relative to a peculiar fluid/tissue. This large spectrum of data demonstrates how the application of these methodologies on a variety of model-systems result in a “concerted effort” for a better understanding of the molecular basis of that disorder. A chapter is also dedicated to show the recent advances of CE-MS, a valuable analytical tool for the analysis of complex protein mixtures, which is complementary to LC-MS. Although only a few of these advances are still focused on respiratory diseases, their potential for delivering improvements in this area is clearly evident.

## 3. Proteomics of Lung Cancer

Lung cancer is a major public health issue and the leading cause of cancer-related deaths worldwide. Being that the clinical outcome of lung cancer patients is still poor (despite advancements in treatments, such as surgery, chemotherapy and radiotherapy), the need to detect this disorder at an early stage is certainly great. There is no doubt that improvements of the current diagnostic methods (*i.e*., computed tomography and chest X-ray) and efforts aimed at the identification of effective biomarkers with a systemic relevance to this disease would be expected to provide a substantial contribution toward increasing the survival rate of lung cancer patients. In this respect, clinical proteomics has emerged as an approach with an eye toward looking further ahead into disease mechanisms in which proteins play a major role. 

### 3.1. Cell Cultures

If *in vitro* culture-based cells are the most widely used model system for the identification of potential biomarkers for the early detection and prognosis of lung cancer, iTRAQ (isobaric tags for relative and absolute quantitation) is one of the most interesting strategies for the measurement of protein expression level [25,26,27,28,29]. 

With this method, the tryptic peptides obtained from the sample under investigation are labeled with a tag that is designed to fragment in MS/MS in such a way that mass information from one of the molecule regions (the “reporter” section) can provide quantitative information concerning the relative amount of the peptide in the sample [30,31]. 

This versatile approach was applied (i) by Keshamouni *et al.* [25] to human lung adenocarcinoma cell line A549; (ii) by Eriksson *et al.* [26] to a small lung cancer cell line, sensitive to the cytotoxic drug, doxorubicin (H69), and to its isogenic doxorubicin-resistant parental cell line (H69AR); (iii) by Chenau *et al.* [27] to a human non-small lung adenocarcinoma cell line of bronchoalveolar origin (H358) with a homozygous deletion of p53; (iv) by Chiu *et al.* [28] to investigate lung cancer cell lines with different invasive and metastatic capabilities (CL1-0 and CL1-5 cells); and (v) by Haura *et al.* [29] for the identification of a protein in a system in which it would not be observed, because of its low-abundance, due to sequestration by an endogenous bait protein in the sample and/or suppression by the high quantity of the bait. 

Works on tumor progression and metastasis, aimed at investigating the role of transforming growth factor-β (TGF-β) in inducing the epithelial to mesenchymal transition (EMT) of epithelial cells, demonstrated, for the first time, that this process in A549 cells results in a migratory and invasive phenotype [25].

In fact, a quantitative differential analysis on TGF-β treated and untreated cells revealed the overexpression, in the former group, of transglutaminase 2 and β1 integrin. This finding was of particular interest, since transglutaminase 2 is characterized by a critical regulatory activity, being implicated in the transformation of latent to active TGF-β [32,33]. In its turn, the upregulation of β1 integrin was previously found to be associated with increased invasion of human lung, breast and ovarian cancer cells [34,35] and to be also required for TGF-β-induced EMT in mammary epithelial cells [36].

The study aimed at demonstrating the primary resistant mechanism to anthracyclines in tumor cells, evidencing the role of a few key-proteins [26]. In particular, Serca 2, a Ca^2+^ pump with ten transmembrane segments located in the endoplasmic reticulum (ER) membrane and responsible for pumping Ca^2+^ into the ER lumen, was under-expressed in the cell line resistant to doxorubicin. The Ca^2+^ levels in the ER were previously shown to play an important role in the cell ability to undergo apoptosis [37]. On the basis of these findings, it was hypothesized that the downregulation of the enzyme responsible for the influx of Ca^2+^ into the ER contributes to the resistance phenotype of H69AR cells. Further, the finding of plectin, vimentin and moesin among the over-expressed proteins in the resistant cell line suggests a more rigid structure of these cells, making them less sensitive to environmental stress.

Particularly intriguing were the results obtained from the quantitative secretome analysis performed on H358 cells. This is a human non-small lung adenocarcinoma cell line of bronchoalveolar origin with a homozygous deletion of p53, a major tumor suppressor protein very frequently mutated and/or inactivated in cancer cells [38]. This proteomic analysis evidenced that the p53 deficient cells overexpressed a protein belonging to the fibroblast growth factor family (FGF-19) that binds the FGF-4 receptor only. This finding was very interesting, since this protein was previously shown to be overexpressed in many tumors and to be implicated in tumor progression [39]. 

Upon investigating lung cancer cell lines with different invasive and metastatic capabilities (CL1-0 and CL1-5 cells), retinal dehydrogenase I (ALIA1), peroxiredoxin-I (PRDX1) and nidogen-1 (NID-1) were found to be higher in CL1-0 and collagen alpha-1 (VI) chain (COL6A1), metalloprotease inhibitor 1 (TIMP1), urokinase-type plasminogen activator (uPA) and alpha-1-antitrypsin (AAT) were higher in CL1-5 [28]. The elevation of the TIMP-1 concentration was previously found to promote cancer progression, enhancing the proliferation of endothelium and angiogenesis [40], and the µPA system of plasminogen activation was correlated with invasion and metastasis [41]. On the other hand, elevated serum levels of AAT have also been observed in numerous cases of malignant diseases and different cancers [42,43]. In its turn, COL6A1, a major component of extracellular matrix (ECM) involved in the organization of fibronectin and other types of collagen (including types I and IV), was shown to be implicated in cell migration and differentiation [44]. On the basis of these data, a pathway analysis that showed how COL6A1 is directly regulated by the proteolysis of metalloproteases (MMP-1 and MMP-7) in ECM could be performed. These results also proved the metastatic abilities of COL6A1, a finding that had never been previously reported. 

The versatility of iTRAQ reagents was successfully demonstrated by applying it to streptavidin-hemagglutinin (SH)-tagged versions of epidermal growth factor receptor (EGFR), expressed in two cancer cell lines (HCC827 and PC9 cells) via retroviral transduction, with the aim of identifying the EGFR core protein complexes [29]. It demonstrated that ubiquitin-associated and SH3 domain-containing protein B (UBS3B), a member of the EGFR core complex, was indeed present in both cell lines, although, due to its markedly reduced quantities, it could not be detected by standard procedures. 

To identify the phosphorylation sites in human non-small lung cancer (NSCLC) cells, Zhang *et al.* [45] focused primarily on the development of a new method in this area. Their strategy, consisting in a combination of protein immunoprecipitation and nano-LC-MS/MS, allowed for the identification of 30 phosphorylation sites, including 12 tyrosines (pY), 12 serines (pS) and six threonines (pT). In addition, upon scanning phosphorylation of EGFR across 31 lung cancer cell lines, they observed that higher stoichiometry of three phosphorylation sites was statistically correlated with EGFR sensitizing mutations. Similarly, higher amounts of phosphorylation on three sites correlated with the sensitivity of erlotinib, an inhibitor of the tyrosine kinase receptor. Five phosphorylation sites on EGFR from the HCC827 cell line, which were inhibited by erlotinib in a concentration-dependent manner, were also identified.

The characterization of phosphorylated proteins is indeed essential for the understanding of lung cancer development, since phosphorylation is a key process in tumor progression for many diseases. In this context, a significant implementation of a human lung cancer phosphoproteome profile was provided by a number of recent articles [46,47,48]. The first large-scale survey of tyrosine kinase activity in lung cancer was that performed by Rikova *et al.* [46], who characterized tyrosine kinase signaling across 41 NSCLC cell lines and over 150 NSCLC tumors. Their approach was particularly interesting, because it provided insight into cancer biology, not available at the chromosomal and transcriptional levels. Known oncogenic kinases (such as EGFR and c-Met), as well as novel anaplastic lymphoma receptor tyrosine kinase (ALK) and proto-oncogene tyrosine-protein kinase ROS (reactive oxygen species) fusion proteins were identified. In addition, other activated tyrosine kinases, such as platelet-derived growth factor receptor *α* (PDGFR*-α*) and discoidin domain receptor tyrosine kinase 1 (DDR1), not previously shown in the genesis of NSCLC, were also identified. 

The two cell lines with different metastatic abilities (CL1-0 and CL1-5) have been investigated also by Wu *et al.* [47] with the aim of identifying new tyrosine phosphorylation sites involved in the metastatic process. Based on their label-free quantitative approach, a total of 36 P-Tyr (30 P-Tyr with higher levels in CL1-5 cells and six P-Tyr with higher levels in CL1-0 cells) were considered to be associated with metastasis. Seven of these proteins appeared of great interest, because they had never been reported before to be associated with lung cancer metastasis. A protein-protein interaction network analysis of these altered proteins revealed that 11 proteins were linked to a network containing EGFR, tyrosine-protein kinase (c-Src), transcription factor Myc (c-Myc) and signal transducer and activator of transcription protein (STAT), which are known to be related to lung cancer metastasis. Yu *et al.* [48] focused on the large-scale study of human lung cancer A549 cell phosphoproteome that had never been analyzed before. Interestingly, most (68%) of the 337 phosphorylation sites (on 181 phosphoproteins) identified in this work appeared to be novel, no matches being found with the known sites contained in the lung cancer cell database. From among these proteins, Yes-associated protein 1 (YAP1), phosphorylated at Ser-127, was observed in three different cell lines. The fact that its expression resulted in clearly decreased in cancer cells and tissues as compared to controls suggested YAP1 to be a promising lung adenocarcinoma biomarker.

Two additional recent articles [49,50] focus on the proteomics of A549 cells. Luo *et al.* [49] analyzed the secretome of this cell line using an approach in which intracellular contaminations were minimized, as assessed by the fact that more than 85% of proteins were accepted as being secretory and around 77% as being extracellular or membrane-bound. The list of qualified secretome included cathepsin D, a candidate that had already emerged as a potential lung cancer biomarker from the analysis of M-BE (a SV40T-transformed human bronchial epithelial cell line) cell secretome [51]. Moreover, findings that had never been reported before concerned C4b-binding protein (C4BP), a protein that controls the classical pathway of complement activation. It was found to exhibit a strong power of discrimination between normal and lung cancer sera, and C4BP serum levels were associated with tumor staging. 

Sitek *et al.* [50] described a label-free differential proteomic analysis performed on the A549 cell line, treated or not with TGF-β. This approach allowed for the detection of 202 differentially expressed proteins. 

A large-scale comparative molecular mapping of microvascular endothelium in human NSCLC tissues and adjacent-normal lung tissues was performed by Park *et al.* [52] to identify lung cancer-related endothelial cell (EC)-selective proteins. This material was isolated from five patients with lung cancer by using CD31 antibody, an endothelial marker widely used to enrich ECs in various tissues [53]. The potential EC-enriched candidate biomarkers included nicotinamide adenine dinucleotide NADH dehydrogenase (ubiquinone), 1 alpha subcomplex 5 (NDUFA5), peroxiredoxin 4 (PRDX4) and thymopoietin (TMPO), which were highly expressed in cancer cells compared to normal ones. Furthermore, COPG, a subunit of the heptameric protein complex coatomer protein I (COP), was found to be highly and specifically expressed in cancer-derived ECs.

Phosphoproteome was also investigated by Rodriguez-Ulloa *et al.* [54] under a different point of view. They performed, for the first time, a proteomic analysis on NSCLC treated with proapoptotic peptide-based drug (CIGB-300). This is a pro-apoptotic peptide-based drug that abrogates the phosphorylation mediated by casein kinase 2 (CK2), a protein overexpressed in a variety of solid and hematopoietic tumors [55,56]. The proteomic profile in response to the treatment with CIGB-300, analyzed by 2DE and 2D-LC-MS/MS, revealed, in particular, the instability of nucleolar phosphoprotein B23 (B23/NPM). The effect of this drug was, in fact, to induce the partial degradation of B23 and to impair the ribosomal biogenesis and translation process. These data confirmed that this cyclic peptide may lead to apoptosis by blocking the CK2-mediated phosphorylation on B23/NPM with subsequent interference with the nucleolar assembly. CIGB-300 was also able to modulate other proteins related to metastasis, including thymidine phosphorylase (TP).

As shown in a couple of recent articles [57,58], also NSCLC subtypes have been used for the discovery of putative lung cancer biomarkers. 

Planque *et al.* [57] have worked on NSCLC cell lines of differing origin, *i.e*., (i) adenocarcinoma, H23; (ii) squamous cell carcinoma, H520; (iii) large cell carcinoma, H460; and (iv) small cells, H1688. To avoid complexity and to have more chances for lung cancer biomarker discovery, cells were grown in serum-free medium. From among the proteins identified, tumor necrosis factor-α-converting enzyme (ADAM-17), soluble tumor necrosis factor-receptor type I (sTNF RI), pentraxin 3; osteoprotegerin and follistatin, whose level was higher in the NSCLC of patients compared to controls, were considered putative new lung cancer biomarkers, also on the basis of the fact that each of them had been previously associated with other types of human cancers.

By analyzing the proteome of the conditioned media of QU-BD and Mehr-80, two subtypes of NSCLC that had never been investigated before, Yousefi *et al.* [58] found that stathmin, vimentin, epidermal fatty acid-binding protein, IL-25, transgelin-2, chloride intracellular channel 4 (CLIC4) and stress-induced phosphoprotein I were overexpressed in lung cancer. These proteins should be further investigated as possible biomarkers of large cell lung carcinoma (LCC).

The aim of the following research was to clarify the paracrine mechanisms involved in the crosstalk between human adipose tissue-derived mesenchymal stem cells (hASCs) and cancer cells. Shin *et al.* [59] characterized the secretome of lysophosphatidic acid (LPA)-conditioned medium by shotgun proteomics. TGF β-induced protein ig-h3 (β ig-h3) was shown, for the first time, to play a pivotal role as an extracellular adhesion molecule, which stimulates the proliferation of A549 lung adenocarcinoma cells *in vitro.* A variety of extracellular proteins, including periostin, IL-8, insulin-like growth factor-binding protein 3/6 and several proteases/protease inhibitors were also identified as LPA-induced secreted proteins.

### 3.2. Serum/Plasma

The fact that almost all body cells communicate (either directly or through tissues/biological fluids) with the plasma and release (upon damage or death) at least a part of their contents into the bloodstream makes human plasma one of the most important sources of information for clinical proteomics. However, given the complexity of this matrix and the wide range of protein concentrations in plasma, only comprehensive systems characterized by a high resolution and wide dynamic range can be applied to achieve a large-scale analysis of plasma proteome. A variety of attractive procedures [60,61,62,63,64,65,66] that could revolutionize current methods for the discovery of new disease-associated protein markers are presented below. 

Fujii *et al.* [60] applied a µLC system coupled to a linear ion-trap mass spectrometer (µLC-2-D ITMS) with a high scan speed to plasma samples (human serum albumin- and Immunoglobulin G-depleted) from healthy subjects and lung adenocarcinoma patients. Low-abundance proteins, of great clinical importance, because they are directly correlated with the progress of various diseases, could be identified. 

An original method consisting in intact protein fractionation followed by chromatographic/ electrophoretic analysis of fractions was developed by Faca *et al.* [61] to achieve in-depth analysis of serum and plasma proteomes. In a few words, pooled serum samples (from healthy controls and newly diagnosed subjects with lung cancer) were depleted of abundant proteins and fractionated by a 2-D LC system consisting of anion-exchange and reversed-phase chromatography. Each fraction was divided into two aliquots, one of which was submitted to shotgun LC-MS/MS and another further resolved on SDS-PAGE. In their turn, the gel bands from this latter step were digested with trypsin and analyzed by MS. Based on the total number of proteins identified and on the representation of specific proteins in individual fractions, they could demonstrate that increased sample fractionation resulted in an increased depth of analysis 

To study the altered pattern of protein glycosylation in cancer, Zeng *et al.* [62] developed a label-free method for a glycoproteome analysis of a large set of NSCLC pooled sera that included adenocarcinoma, squamous cell carcinoma) and matched control sera. Their method, based on glycoprotein capture and enrichment, allowed for the isolation of *N*-linked glycosylated peptides and their identification by LC-MS/MS. This comparative analysis led to a near complete separation of case and control pools. Further application of this method on a similar set of pooled NSCLC sera led the same authors [63] to estimate, via spectral counting, the relative abundance of proteins across these pools. More inflammatory response-related proteins were found to be differentially abundant in adenocarcinoma case pools compared to squamous cell carcinoma pools. In their turn, these latter showed a greater response in plasma lipid physiology with more differentially abundant proteins involved in molecular transport. 

Applying the same label-free nano-LC-MS/MS method indicated above, Ueda *et al.* [64] obtained a comprehensive peptidome profiling of lung adenocarcinoma serum from fractions that had been enriched by one-step size exclusion chromatography (M_r_ range: 1,000–5,000). This approach allowed for the identification of 12 serum peptides as reliable candidate biomarkers for both early detection and tumor staging of lung cancer. Eight of them were fragments derived from fibrinopeptide A, the product of fibrinogen α (FIBA) cleavage; two were generated from apolipoprotein APOA4; one was a fragment from limbin (LBN) and another from amiloride-sensitive cation channel 4 (ACCN 4).

The newly-developed method of Torsetnes *et al.* [65] (that combined selective sample preparation by immunoextraction and identification of signature peptides by LC-MS) was applied to serum samples of SCLC patients. Total progastrin-releasing peptide (ProGRP) was identified as a marker in sera at clinically relevant levels. This method also proved ProGRP isoform occurrence in these sera and differentiated, for the first time, between isoform 1 and 3. From the values of these variants, the amount of ProGRP isoform 2 could also be indirectly calculated. 

The attractive method proposed by Oh *et al.* [66] consisted in the combination of longitudinal proteomic analysis with a novel graph-based computational method. In brief, the approach utilizes longitudinal changes of candidate proteins that are located in close proximity or share similar signaling pathways with other biomarkers implicated in the disease that may not necessarily be robust enough for clinical practice. By measuring the closeness between candidate proteins and regularization proteins identified from prior knowledge of the disease process, also a search from a limited sample size may produce relevant biomarkers.

This approach was applied to 26 serum samples collected longitudinally before and during the course of fractionated irradiation treatment of matched-control locally advanced NSCLC patients with and without clinically proven radiation pneumonitis (RP), the manifestation of radiation-induced lung injury. On the basis of the proposed methodology, α-2-macroglobulin (α-2M) was unambiguously ranked as the top candidate protein potentially predictive of early RP onset.

The involvement of serum amyloid A (SAA) in cancer pathogenesis was investigated by Sung *et al.* [67] on crude serum and plasma samples of lung adenocarcinoma and lung cancer of other histological types. Samples were fractionated by SDS-PAGE, gel bands analyzed by LC-ESI-MS/MS and both SAA1 and SAA2 isomers identified. The finding of the higher concentrations in the blood of lung cancer patients than in subjects with other respiratory diseases or other cancers suggested the SAA level in some lung cancers to be a useful differential diagnostic marker. Interestingly, co-culturing lung cancer cells with macrophages resulted in increases of IL-1β and IL-6, which, in turn, stimulate lung cancer cells to induce SAA1/2 production. In conclusion, a lung cancer cell itself expresses and secretes SAA1/2 upon interaction with and stimulation by immune cells residing in the tumor microenvironment. These proteomic data were validated by ELISA analysis.

A linear ion trap quadrupole (LTQ)-Orbitrap platform was applied by Qin *et al.* [68] to pre-diagnostic sera as a new approach to determine whether a set of antigens (consisting of annexin I, P-glycoprotein 9.5 and 14-3-3 theta), previously found to be associated with autoantibodies at the time of diagnosis, discriminates between cases and controls before the onset of symptoms. The occurrence in lung cancer sera of the autoantibodies to antigens defined above and the discovery of laminin receptor-like 1 as a novel lung cancer antigen suggested the potential utility of this approach to diagnose lung cancer before the onset of symptoms. The report by Yildiz *et al.* [69] illustrates a matrix-assisted laser desorption ionization (MALDI)-MS procedure to identify a proteomic signature, directly obtained from unfractionated serum, aimed at distinguishing lung cancer cases from matched controls. From a blinded test set of matched samples, a serum protein signature consisting of seven peptides was found to be associated with lung cancer. 

### 3.3. Tissues

In pursuit of reports dealing with the proteomics of tissues for the biomarker discovery of lung cancer, the article by Marko-Varga *et al.* [70] is worth reading, because it illustrates, with great accuracy, the principles and concepts of different approaches for the discovery of biomarker candidates. Both qualitative and quantitative techniques for determining the specific patterns of proteins detected in tissue and/or biofluids of experimental animal models are described. Human respiratory tract following life-long cigarette smoking in the context of the development of COPD and/or cancer is also documented. 

The technical feasibility of a shotgun LC/MS-based method for the global proteomic study of formalin-fixed paraffin-embedded (FFPE) materials was demonstrated by Kawamura *et al.* [71]. Archived clinical FFPE specimens of seven patients with stage IA lung adenocarcinoma without lymph node involvement and another six more advanced stage IIIA subjects, with the spread to lymph nodes, were analyzed to characterize protein expression that could reflect the clinical stages of the disorder. The reliability of this technique was confirmed by multiple-reaction monitoring MS on a subset of these proteins [72]. 

iTRAQ labeling, described above, and the HPLC-Orbitrap-MS platform were used by Li *et al.* [73] to study the expression of tumor-associated proteins and their potential roles in lung adenocarcinomas. All tumors and tumor-matched normal lung tissues used for their investigation were fixed in formalin and embedded in paraffin prior to proteomic analysis. MUC5B, a high-molecular weight, heavily-glycosylated protein, showed significant changes in tumor tissues. The validation of its aberrant expression using immunohistochemistry suggested for this protein a role as a potential biomarker in the detection of adenocarcinomas.

Wei *et al.* [74] developed a new platform to define the proteomic profiles of NSCLC xenografts using a set of ten human tumors [five adenocarcinoma (ADC) and five squamous cell carcinoma (SCC)] that were directly introduced into severely immune deficient mice. The identity and quantity of proteins in the resulting xenograft tumors were obtained from proteomic analysis (in addition to standard histology and immune-histochemistry). Although this study was not aimed at identifying differentially expressed proteins, a comprehensive panel of intermediate filament keratin proteins that could represent a distinctive proteomic signature associated with the NSCLC subtypes was observed. These results confirmed the potential of the method to discover and link tumor molecular markers with the cancer phenotype.

With the aim of searching biomarkers for the early detection of lung squamous cell carcinoma (LSCC), Zeng *et al.* [75] applied iTRAQ in combination with 2D-LC-MS/MS to identify differential proteins among different types of tissues (normal bronchial epithelium; squamous metaplasia; atypical hyperplasia; carcinoma *in situ* and invasive LSCC). The combination of glutathione S-transferase P1, heath shock protein beta-1 and creatine kinase brain-type was found to perfectly discriminate normal bronchial epithelium from neoplastic/preneoplastic lesions. For the first time, these three proteins were shown to be novel potential biomarkers for the early detection of LSCC. 

Kikuchi *et al.* [76] were the first to use a standardized label-free shotgun proteomic analysis for in-depth tissue protein profiling of the two major subtypes of NSCLC and normal lung tissues. From the analysis of pooled human samples of SCC, adenocarcinoma and control specimens, they identified new pathways and new differentially expressed proteins (*i.e*., the p21 activated kinases) of potential interest as diagnostic biomarkers. Multiple reaction monitoring (MRM)-MS confirmed the upregulation of these proteins.

An interesting method (consisting in the combination of 2DE and highly sensitive differential gel electrophoresis (DIGE) saturation labeling for the analysis of limited amounts of microdissected material), established by Poschmann *et al.* [77] to monitor protein changes in SCC tumor progression, revealed that heat-shock protein 47 (HSP47) and a group of cytokeratins were significantly co-regulated in SCC.

### 3.4. Pleural Effusions

Pleural effusion is produced continuously at the parietal pleural level and reabsorbed through the lymphatic system. In a number of disorders, including cancer, it accumulates, since the rate of fluid formation exceeds the rate of removal. While being rich in proteins, either derived from the circulation or locally released by inflammatory or epithelial cells, most of them have not been identified, yet. A database useful for a better understanding of the mechanisms involved in lung cancer pathogenesis was generated for the first time by Tyan *et al.* [78], who applied 2D-nano-LC-MS/MS to pleural effusions of forty-three patients with lung adenocarcinoma.

Soltermann *et al.* [79] applied shotgun MS on malignant pleural effusions of five patients with advanced lung adenocarcinoma and five free of tumor (controls). They aimed at: (i) verifying whether known and/or novel potential N-glycoprotein biomarkers could be detected in this fluid; and (ii) validating protein identification by clinical chemistry and immunocytochemistry. In addition to the already validated markers (*i.e*., CA-125, CD44, TTF-1 and SP-A), novel potential candidates implicated in metastatic processes (periostin, multimerin-2, CD-166 and lysosome-associated membrane glycoprotein-2 (LAMP-2)) were identified. They concluded that, while being complex, the pleural effusion model is a valid source of secreted proteins. Being simpler than lung cancer tissue, in terms of cellular composition, proteins in the microgram to nanogram/milliliter range could be detected.

### 3.5. Urine

Based on the evidence that urinary proteome can be highly informative also in non-urogenital diseases, Zhang *et al.* [80] have compared the urinary proteomic profiles of normal individuals and NSCLC patients with the purpose of identifying biomarkers for NSCLC diagnosis. From among the unique proteins identified by 1DE and nano-LC-MS/MS, clusterin, kallikrein, gelsolin, leucine-rich α-2-glycoprotein and α-1-antichymotrypsin were related to lung cancer. In particular, the finding of higher levels of α-1-antichymotrypsin in NSCLC patients, compared to controls, confirmed that urine could be a non-invasive source of candidate biomarkers of NSCLC.

## 4. Proteomics of Asthma

Asthma is a chronic heterogeneous disorder characterized by airway obstruction and inflammation that varies in severity across the spectrum of the disease. Being a growing global health problem, tools that allow its early diagnosis, monitoring and follow-up would offer great promise for understanding its pathophysiology. The following paragraphs demonstrate that the analysis of a variety of fluids may allow for the identification of specific biomarkers for asthma.

### 4.1. Plasma/Serum

The iTRAQ strategy widely discussed above for investigating cell proteomics in lung cancer (see [25,26,27,28,29] and [73,75]) was used also by Singh *et al.* [81] to determine changes in plasma proteome of mild atopic asthmatic individuals undergoing allergen inhalation challenge. Blood samples were collected from adults with mild, allergic asthma, both early (ERs) and dual responders (DRs), prior to and 2 h after the inhalation challenge and analyzed with pooled controls. Although no differences between pre- and pro-challenge samples were observed, the study was the proof of principle that this technology was sensitive enough to uncover differences in the human plasma proteome between two endotypes of asthma. At pre-challenge, α-1B-glycoprotein, inter-α(globulin) inhibitor H4 isoform 2 precursor, transthyretin and fibronectin 1, isoform 2 preproprotein (FN1), were differentially expressed between selected ERs and DRs. In particular, the ability of FN1 to discriminate ERs from DRs was novel in asthma.

### 4.2. Induced Sputum

Based on the fact that the fluid phase of induced sputum is a rich source of lung proteins, the proteome of induced sputum in healthy subjects and asthmatic patients with and without exercise-induced bronchoconstriction (EIB) was explored by Gharib *et al.* [82] by applying shotgun proteomics. Induced sputum proteome was found to be rich in proteins involved in defense response, immunity, protease inhibitory activity and inflammation. Among these, while α_1_-antitrypsin (SERPIN1) was significantly overexpressed, the founding member of the secretoglobin superfamily (SCGB1A1) was under-expressed in asthmatic patients. This proteomic analysis also allowed for the identification of subjects susceptible to EIB on the basis of a significant increase of protein C3, a member of the phylogenetically conserved complement system, and of HPX, an acute-phase heme-binding protein.

### 4.3. Exhaled Breath Condensate

Because of its direct origin from the airways, exhaled breath condensate (EBC) reflects the physiological state of the respiratory tract at a specific moment, thus being an excellent matrix to assess airway inflammation. 

Taking advantage of these characteristics, Bloemen *et al.* [6] examined the EBC of asthmatic and non-asthmatic children to understand whether these two groups could be discriminated on the basis of their proteomic profile. The application of nano-LC and MALDI-time of flight (TOF)/TOF resulted in the selection of a pattern of differentially expressed peptides derived from exhaled proteins that was characteristic for the EBC of the partially and uncontrolled asthmatic patients. This distinguished the two groups. Although the low abundance of these peptides prevented their identification, this was a step forward in the detection of proteins present in the EBC of asthmatic subjects. 

However, the measurement and identification of proteins in the EBC remains an area where increasing standardization will be required. In order for EBC to reflect accurately the composition of the lining of the lower airways, upper airways contaminants need to be kept at a minimum. For example, mouth rinsing, voluntary swallowing of saliva and a saliva trap may decrease contamination. In other words, while being simple, safe and completely noninvasive, EBC collection has yet to be standardized. A few reports recently published [83,84,85] indicate the benefits, pitfalls and possible future development of this approach.

### 4.4. Bronchoalveolar Lavage Fluid

Despite the publication of numerous reports dealing with the characterization of proteins in bronchoalveolar lavage fluid (BALf) in various pulmonary disorders [24,86,87], the comparative proteomic analysis of both allergic asthmatic patients and healthy volunteers had never been investigated. The first differential study on the BALf of these individuals was performed by Wu *et al.* [88], who produced, for the first time, a comprehensive BALf protein database. Abundant proteins were depleted by immunoaffinity chromatography and the remaining proteins separated by SDS-PAGE and identified by nano-LC-MS/MS. Being that asthma is a complex disorder, it is no wonder that the proteins identified were involved in numerous functions, including proteolysis, inflammatory responses, cell adhesion, cell mobility and proliferation, metabolism and signal transduction. Chemokines and cytokines and a variety of MMPs were shown to be distinctly elevated in subjects after segmental allergen challenge. Other highly overexpressed proteins included pulmonary surfactants and LPLUNC1.

## 5. Proteomics of Cystic Fibrosis

Being that airway inflammation and recurrent infections leading to respiratory failure are the major causes of morbidity and mortality among cystic fibrosis (CF) patients, they obviously represent the main focus of clinical research. Thus, useful tools towards the identification of biomarkers for the diagnosis/prognosis of this disorder may be the characterization of proteins from different sources and the study of their interactions within the lung microenvironment. The paragraphs below show the most recent reports on proteomic research in this field.

### 5.1. Cell Cultures

The peculiarity of the novel approach developed by Pollard *et al.* [89] consisted in its ability to identify proteins whose abundance and rate of biosynthesis are modified by a disease status, CF in their specific case. In a few words, 2DE allowed for the observation of a change in silver stain intensity of 20 (out of 194) spots of cultured CF lung epithelial cells (IB3-1) in comparison with daughter cells repaired by gene transfer with wild-type cystic fibrosis transmembrane conductance regulator (CFTR) (IB3-1/S9). However, the simultaneous measurement (with ^35^S-labelled methionine) of *de novo* biosynthetic rates of all 194 proteins in both cell types resulted in the identification of an additional 31 CF-specific proteins. The conclusion was drawn that the proteome-wide measurement of the individual rates of protein biosynthesis could be used to obtain significant disease-specific kinetic information in the high abundance proteome of CF. In particular, only keratin 18, 3-hydroxy-3-methylglutaryl-CoA synthase I, ubiquitin carboxy-terminal hydrolase L1, translationally controlled tumor protein, guanylate cyclase activator 1C and heat shock proteins 27, which distinguished disease from the control, were found to overlap between the two groups. 

### 5.2. Serum

In an effort to obtain more insights into the pathogenesis of CF, immunodepleted sera of CF patients and of healthy CF and non-CF carriers were analyzed by Charro *et al.* [90] with a combination of 2D-PAGE and shotgun LC-MS/MS for the identification of medium- and low-abundant proteins. This comparative study resulted in the finding of deregulated proteins involved in tissue remodeling, complement system dysfunction with consequent impairment on defense mechanisms and chronic inflammation, nutritional imbalance and *P. aeruginosa* colonization. The confirmation of these altered biological processes as implicated in the pathogenesis of CF by reports from other authors was of great importance in corroborating the reliability of these results. In particular, members of the apolipoproteins family were found to be deregulated in CF patients. Heat shock 70 kDa protein 5 and the multifunctional enzyme, NDKB (whose functions account for an ion sensor in epithelial cells, pancreatic secretion, neutrophil-mediated inflammation and energy production), were identified exclusively in the CF group.

## 6. Proteomics of Chronic Obstructive Pulmonary Disease

Early correct diagnosis of chronic obstructive pulmonary disease (COPD) may be very difficult, this disorder being very heterogeneous and potentially representing several disease phenotypes. It is a common opinion that the finding of molecular markers of a specific disease phenotype would strongly contribute to speeding up its diagnosis and making the treatment more effective. One of the first works of proteomic research on COPD was carried out in our laboratory as a pilot study on the sputum of 56 individuals divided into five groups (non-smokers, with no airway disease; healthy smokers, with neither airflow obstruction nor mucus hypersecretion; chronic bronchitis, with smokers complaining about cough and phlegm, but without airflow obstruction; a COPD group, with smokers with airflow obstruction, but no emphysema; and COPD and emphysema (E), subjects with both airway obstruction plus significant emphysema) [3]. Capillary LC-electrospray ionization-quadrupole (ESI-Q)-TOF allowed for the identification of proteins that had never been described before in sputum. In particular, mucins were the major glycoconjugate components of sputum together with the protease/antiprotease cascades and complementary serine endopeptidase inhibitors (SERPIN 1 and SLPI). The innate immune system PLUNC proteins, especially important for protection against Gram-negative bacteria, were differentially expressed among groups, and histone 4 was identified only in the COPD and E group. 

The application of LC-MS/MS to the EBC from healthy smokers and COPD patients, also performed in our laboratory [8], resulted in the production of a “fingerprint” characteristic for each group of subjects investigated. Several inflammatory cytokines, type I and II cytokeratins, two SP-A isoforms, calgranulin A and B and α_1_-antitrypsin were detected and validated. 

Based on the observation that the disease phenotype in COPD appears to be different between men and women [91,92], Kohler *et al.* [93] analyzed the BALf of patients in the early disease stage with a special focus on the differentiation between the genders. 2D-DIGE and nano-LC-MS were applied on samples from smokers with normal lung function and patients with mild-to-moderate COPD, matched in terms of age and gender. Clear differences were observed in proteome alterations between men and women. In fact, while the alveolar macrophages of females displayed significant phenotypic alterations due to the development of COPD, no alterations were observed in men. Being that cathepsin B (under-expressed), ATP synthase (overexpressed) and chaperonin (overexpressed) were highly and significantly altered, these were considered the most prominent disease marker candidates. These results contributed to the elucidation of putative molecular mechanisms underlying gender-specific differences in the pathophysiology of this disease. More specific details on potential COPD markers can be found in [22].

## 7. Proteomics of Other Respiratory Diseases

### 7.1. Arterial Hypertension

Abdul-Salam *et al.* [94] have recently applied label-free nano-LC-MS/MS to investigate, for the first time, the changes of protein expression in the lung of patients with pulmonary arterial hypertension (PAH), a disorder of vascular remodeling that causes increased resistance to pulmonary blood flow. Proteins that were found to vary in abundance between PAH and control lung tissues were RAGE, annexin A3, CLIK 1, CLIK 4, periostin, transcriptional activator protein Pur-a (overexpressed) and haptoglobin (under-expressed). Most of the more abundant proteins in PAH were associated with roles in cell growth, proliferation, intracellular trafficking and signaling.

### 7.2. Graft Dysfunction

As already discussed in previous paragraphs, BALf is a source of potential surrogate markers for various lung diseases, including chronic graft dysfunction (CGD), the major cause of morbidity and mortality in post-transplant patients. This fluid has been used by Kosanam *et al.* [95] to discover novel biomarkers of lung transplant patients with or without CGD. A novel protocol, consisting in an initial step based on the separation of the proteome by size exclusion chromatography followed by LC-MS/MS separation of the tryptic digests of each fraction, allowed them to obtain the largest dataset of BALf proteins ever published. Increased production of MMP-9, myeloperoxidase and mucins was observed in patients with CGD.

A schematic summary of different matrices investigated and of the methods applied for the proteomic profiling of pulmonary diseases is presented in Table 1.

**Table 1:** List of different matrices investigated and of methods applied for the proteomic profiling of pulmonary diseases considered in this report. iTRAQ, isobaric tags for relative and absolute quantitation; 1DE, one-dimensional gel electrophoresis; NSCLC, non-small cell lung cancer; BALf, bronchoalveolar lavage fluid.

Method	Matrix	Reference
Cancer		
iTRAQ-2D-LC-MS/MS	A549 human lung carcinoma cell line	[25]
Isoelectric focusing (IEF);	Human H69 and H69AR small lung cancer cell line	[26]
iTRAQ-1D-LC-MS/MS	H358 human non-small cell lung adenocarcinoma cell line	[27]
1DE; iTRAQ-1D-LC-MS/MS	CL1-0 and CL1-5 lung cancer cell lines	[28]
iTRAQ-1D-LC-MS/MS	HCC827 and PC9 cell lines	[29]
1DE; 1D-LC-MS/MS	NSCLC cell lines	[45]
	A549 human lung carcinoma cell line	[49]
1D-LC-MS/MS	NSCLC cell lines	[46]
	CL1-0 and CL1-5 lung cancer cell lines	[47]
	A549 human lung carcinoma cell line	[48,50,59]
	H23, H520, H460 and H1688 cell lines	[56]
1DE; 2D-LC-MS/MS	NSCLC cell lines	[52]
2DE; 2D-LC-MS/MS	NCI-H125 NSCLC cell line	[54]
2D-LC-MS/MS	QU-DB and Mehr 80 LCC cell lines	[58]
1D-LC-MS/MS	Human plasma	[60]
2D-LC-MS/MS		[61,62]
2D-LC-MS/MS	Human serum	[61]
1D-LC-MS/MS		[62,63,64,65,66,68]
1DE; 1D-LC-MS/MS		[67]
2DE; 1D-LC-MS/MS	Human lung tumor tissues	[70,77]
1D-LC-MS/MS		[71,72]
iTRAQ-1D-LC-MS/MS		[73]
2D-LC-MS/MS		[74]
iTRAQ-2D-LC-MS/MS		[75]
IEF-1D-LC-MS/MS		[76]
2D-LC-MS/MS	Lung adenocarcinoma pleural effusions	[78]
1D-LC-MS/MS		[79]
1DE; 1D-LC-MS/MS	Human urine	[80]
**Asthma**		
iTRAQ-MS/MS; LC-MRM/MS	Human plasma	[81]
1D-LC-MS/MS	Induced sputum	[82]
1D-LC-MS/MS	Exhaled breath condensate	[6]
1DE; 1D-LC-MS/MS	BALf	[88]
**Cystic fibrosis**		
2DE-MS/MS; 1DE-LC-MS/MS	IB3-1 And IB3-1/S9 cell lines	[89]
2DE; 1D-LC-MS/MS	Human serum	[90]
**COPD**		
CapLC-MS/MS	Induced sputum	[3]
1D-LC-MS/MS	Exhaled breath condensate	[8]
1D-LC-MS/MS	BALf	[93]
**“Minor” respiratory diseases**		
1DE; 1D-LC-MS/MS	Human lung tissue	[94]
Size exclusion chromatography (SEC); 1D-LC-MS/MS	BALf	[95]

## 8. Novel Potential Biomarkers Discovered

Acute and chronic inflammation, tissue damage and oxidative stress are parts of the complex procedure in lung cancer. Despite this complexity, the numerous studies presented here, performed on different tissues/fluids, have documented a large set of candidate protein markers with potential clinical relevance. Cancer-associated inflammation was shown to be linked with an increase of serum amyloid A proteins (SAA1 and SAA2 isomers). These proteins, secreted during the acute phase of inflammation, have several roles, including the recruitment of immune cells to inflammatory sites and the induction of enzymes that degrade extracellular matrix. In their turn, the pro-inflammatory cytokines, IL-1β, IL-6 and TNF-α, regulators of SAA1 and SAA2 genes, were also found to be increased in patients with lung adenocarcinoma. 

Oxidative inactivation of anti-proteases and surfactants, membrane lipid peroxidation, mitochondrial respiration, alveolar epithelial injury, remodeling of the ECM and apoptosis are processes that could contribute to the pathogenesis of lung cancer. For example, COL6A1, a major component of the ECM involved in the organization of fibronectin and other types of collagen (including types I and IV), was shown to be implicated in cell migration and differentiation. A pathway analysis performed on the basis of these data demonstrated that COL6A1 is directly regulated by proteolysis of metalloproteases (MMP-1 and MMP-7) in the ECM. These results, while confirming that multiple proteases causing collagen degradation are important in the pathogenesis of lung cancer, proved the metastatic abilities of COL6A1. This finding had never been previously reported. A variety of MMPs (MMP-7; MMP-8; MMP-9 and MMP-20) was shown to be distinctly elevated also in the bronchoalveolar lavage fluid of subjects after segmental allergen challenge. Quantitative analysis of PLUNC in the same individuals showed overexpression also of this family of proteins of the innate immune system (especially important for protection against Gram-negative bacteria). Likewise, PLUNC proteins were found to be differentially expressed in the sputum of subjects with COPD at different levels of severity. Their expression level was in fact positively correlated with the severity of the disease. 

The high levels of AAT found in lung cancer cell lines with high invasive and metastatic capabilities seemed to correlate with the elevated serum levels observed in other numerous cases of malignant diseases and different cancers. Consistent with this observation was the finding that Serpin1 was overexpressed also in the sputum of both COPD and asthma patients. 

Collectively, these results point to a significant overlap of altered biological processes (such as inflammation, airway remodeling, tissue damage and repair and plasma infiltration) implicated in the pathogenesis of pulmonary disorders of “different” origin and may pose a question about the ability of proteomic strategies to differentiate between diseases. This overlap of differentially expressed proteins across different diseases is one of the fundamental problems of assigning a “biomarker” status for a protein found to be over/under-expressed in a disease. The finding of numerous proteins implicated across a number of different diseases makes the notion of a single biomarker to indicate a specific disease more difficult. Potentially, for greater confidence in disease diagnosis or prognosis, it is required that a suite of biomarkers be needed to provide specificity. In other words the question arises of whether the identification of hundreds of candidate biomarkers comes at the price of sacrificing their specificity. This apparent discrepancy may be reconciled with a “harmonization” of results. In fact, given that no single molecule can sufficiently discriminate complex processes without context, the finding of common signatures for several pulmonary disorders contributes to drawing intriguing parallels among them, which result in a better understanding of the disease mechanism. By contrast, the fact that many proteins found in COPD or in lung cancer have not been reported in other chronic lung diseases suggests that the merits of proteomics in hunting down biomarkers with high specificity cannot be under evaluated. The finding of significantly altered levels of cathepsin B, ATP synthase and chaperonin in the BALf of female COPD patients, but not in that of males, while supporting the hypothesis that these proteins were the most prominent marker candidates for this gender only, confirmed the above-mentioned merits of proteomics. 

The comparison of the microsomal proteomic profile of a small lung cancer cell line sensitive to the cytotoxic drug, doxorubicin, with that of its isogenic doxorubicin-resistant parental cell line, revealed that a Ca^2+^ pump responsible for pumping Ca^2+^ into the ER lumen was under-expressed in the resistant cell line. The finding of plectin, vimentin and moesin among the overexpressed proteins in this cell line suggests a more rigid structure of these cells, making them less sensitive to environmental stress. Calcium-binding proteins, such as cathepsins and calgranulins, were overexpressed also in patients with COPD. 

A large-scale study of phosphoproteome in human lung cancer A549 cells allowed for the identification of a new phosphoprotein (YAP1), phosphorylated at Ser127, which seemed to be a promising lung adenocarcinoma biomarker. Its expression was, in fact, clearly decreased in cancer cells and tissues as compared to controls. 

The analysis of the p53-modulated secreted proteins correlated with p53 tumor suppressor function allowed for the identification of the most comprehensive and global secretome list of these proteins to date. Particularly intriguing was the finding that, while many of the overexpressed proteins secreted upon p53 expression prevented tumorigenesis, the p53 deficient cells over-secreted proteins involved in the progression of this event. In this context, the identification of a protein belonging to the fibroblast growth factor family (FGF-19) that binds the FGF-4 receptor only was of great interest. This protein was in fact previously shown to be overexpressed in many tumors and to be implicated in tumor progression [39]. 

Although caution should be placed when depletion/enrichment strategies (which can result in a loss of less abundant proteins) are applied, the isolation of N-linked glycosylated peptides confirmed a few markers (*i.e*., SP-A, a marker also for COPD) and found novel potential candidates, including periostin, multimerin-2, CD-166 and lysosome-associated membrane glycoprotein-2 (LAMP-2). All of them are implicated in metastatic processes. Gelsolin, a putative urinary biomarker, was overexpressed also in lung tissues of healthy smokers and asthmatic patients. 

In summary, it is largely a matter of speculation whether proteomics contributes to the discovery of clinical entities that define and/or predict normal and pathogenic states. It is often the case that these “low hanging fruit” molecules that turn up time and time again in biomarker studies are of a high abundance, e.g., inflammatory proteins, which are symptomatic of aggressive cancers and advanced disease, rather than having etiologic roles in these disorders. These proteins are often multifunctional, making them poor targets for new therapies. Nevertheless, the articles discussed in this report show that proteomics is currently equipped to yield information not possible by other means, e.g., the accurate identification, quantification and localization of low abundance (and post-translationally modified) proteins in various cellular compartments and extracellular space. The repeatability and high throughput of these procedures allow for the performance of studies that are indeed large enough in scope.

A summary of potential lung cancer biomarkers detected in tissues/fluids described in the above paragraphs is shown in Table 2.

**Table 2:** Summary of potential lung cancer biomarkers detected in tissues/fluids considered in this report.

Proteins	Matrix
**Cancer**	
Transglutaminase 2	A549 human lung carcinoma cell line
b1 integrin	
Yes-associated protein 1 (YAP1)	
Cathepsin D	
Transforming growth factor β -induced protein ig-h3 (β ig-h3)	
Periostin	
Interleukin-8 (IL 8)	
Insulin-like growth factor-binding protein 3/6	
Serca 2	Human H69 and H69AR small lung cancer cell line
Plectin	
Vimentin	
Fibroblast growth factor-19 (FGF-19)	H358 human non-small cell lung adenocarcinoma cell line
P53-modulated secreted proteins	
Retinal dehydrogenase (ALIA1)	CL1-0 and CL1-5 lung cancer cell lines
Peroxiredoxin-I (PRDX1)	
Nidogen 1 (NID-1)	
Collagen alpha-1 (VI) chain (COL6A1)	
Matrix metalloprotease 1 (MMP-1)	
Matrix metalloprotease 7 (MMP-7)	
Metalloprotease inhibitor 1 (TIMP1)	
Urokinase-type plasminogen activator (uPA)	
Alpha-1-antitrypsin (AAT)	
Tyrosine-protein kinase (c-Src)	
Transcription factor Myc (c-Myc)	
Signal transducer and activator of transcription protein (STAT)	
C4b-binding protein (C4BP)	
Ubiquitin associated and SH3 domain-containing protein B	HCC827 and PC9 cell lines
NADH dehydrogenase (ubiquinone)	NSCLC cell lines
1 alpha subcomplex 5 (NDUFA5)	
Peroxiredoxin 4 (PRDX4)	
Thymopoietin (TMPO)	
Epidermal growth factor receptor (EGFR)	
c-Met	
Anaplastic lymphoma receptor tyrosine kinase (ALK)	
Platelet-derived growth factor receptor α (PDGFR-α)	
Discoidin domain receptor tyrosine kinase 1 (DDR1)	
Tumor necrosis factor-a-converting enzyme (ADAM-17)	H23, H520, H460 and H1688 cell lines
Soluble tumor necrosis factor-receptor type I (sTNF RI)	
Pentraxin 3	
Osteoprotegerin	
Follistatin	
Stathmin	QU-DB and Mehr 80 LCC cell lines
Vimentin	
Epidermal fatty acid-binding protein	
Interleukin-25 (IL-25)	
Transgelin-2	
Chloride intracellular channel 4 (CLIC4)	
Stress-induced phosphoprotein I	
Fragments derived from Fibrinopeptide A	Human serum
Fragments derived from Apolipoprotein APOA4	
Fragment derived from Limbin (LBN)	
Fragment derived from amiloride-sensitive cation channel 4 (ACCN4)	
Progastrin—releasing peptide (ProGRP)	
α2-macroglobulin (α-2M)	
Serum amyloid A (SAA)	
Interleukin-1β (IL-1β)	
Interleukin 6-6 (IL-6)	
Annexin-I	
P-glycoprotein 9.5	
14-3-3 theta	
Laminin-receptor-like 1	
Cancer antigen 125 (CA-125)	Human lung tissue
Cell-surface glycoprotein 44 (CD44)	
Thyroid transcription factor 1 (TTF-1)	
Surfactant protein A (SP-A)	
Periostin	
Multimerin-2	
Antigen CD 166 (CD-166)	
Lysosome-associated membrane glycoprotein-2 (LAMP-2)	
Glutathione S-transferase P1	Human lung tissue
Heat-shock protein beta-1	
Creatine kinase brain-type	
p21 activated kinases	
Heat-shock protein 47 (HSP 47)	
Various cytokeratins	
Mucin 5B (MUC 5B)	
Clusterin	Human urine
Kallikrein	
Gelsolin	
Leucine-rich α 2-glycoprotein	
α-1-antichymotrypsin	
**Asthma**	
α-1B-glycoprotein	Human plasma
Inter-α (globulin) inhibitor H4 isoform 2 precursor	
Transthyretin	
Fibronectin 1	
Isoform 2	
Preprotein (FN1)	
Alpha-1-antitrypsin (AAT)	Induced sputum
Secretoglobin family A1 member 1 (SCGB1A1)	
Complement component 3 (C3)	
Acute-phase heme binding protein (HPX)	
Matrix metalloprotease 7 (MMP-7)	BALf
Matrix metalloprotease 8 (MMP-8)	
Matrix metalloprotease 9 (MMP-9)	
Matrix metalloprotease 20 (MMP-20)	
Long palate lung and nasal epithelium carcinoma-associated protein 1 (LPLUNC1)	
**Cystic fibrosis**	
Keratin 18	IB3-1 And IB3-1/S9 cell lines
3-hydroxy-3-methylglutaryl-CoA synthase I	
Ubiquitin carboxy-terminal hydrolase L1	
Translationally controlled tumor protein	
Guanylate cyclase activator 1C	
Heat shock protein 27	
Apolipoprotein A-I	Human serum
Apolipoprotein B-100	
Vitamin D binding protein	
Alpha-1-antitrypsin (AAT)	
Mucin 16	
Angiotensinogen	
Vinculin	
Aspartyl-tRNA synthetase	
Alpha-1-antitrypsin (AAT)	Induced sputum
Antileukoproteinase (SLPI)	
Palate, lung and nasal epithelium clone (PLUNC) proteins	
Histone 4	
Several cytokines	EBC
Type I cytokeratin	
Type II cytokeratin	
Alpha-1-antitrypsin (AAT)	
Surfactant protein A (SP-A) isoforms	
Calgranulin A	
Calgranulin B	
Cathepsin B	BALf
ATP synthase	
Chaperonin	
**“Minor respiratory diseases”**	
Chloride intracellular channel protein 1	Human lung tissue
Chloride intracellular channel protein 4	
Periostin	
Haptoglobin	
Transcriptional activator protein Pur-alpha	
Advanced glycosylation end product-specific receptor (RAGE)	
Annexin A3	
Mucins	BALf
Matrix metalloprotease 9 (MMP-9)	
Myeloperoxidase	

## 9. Ten Years of Technological Advances in Respiratory Proteomics

While the combination of gel-based separation techniques (1- or 2-DE) and MALDI-TOF is the technological platform employed in a good number of protocols discussed in this report (see [27,58,69,70,77,81,89,90]), the poor resolution of this strategy often limits its applicability on complex protein mixtures. However, given that the ability to first separate proteins/peptides is fundamental to any identification or further characterization, a progressive refinement in materials and methods was naturally afforded over the last few years for the better characterization of a biological system. In terms of materials, while C_18_-bonded porous silica 3–5 µm-sized particles are the most widely used packing materials, peculiar and highly flexible systems (including monolithic columns (see [26]), self-packed columns (see [52,61,62,63,70]) or trapping columns with specific selectivity for target proteins (see [62,63,79])) have also been frequently applied to extend the potential of methods. Column characteristics (in terms of length and inner diameter) have progressed in parallel with the evolution of materials. In this respect, works have been typically performed with micro- and nano-HPLC columns, whose performance, based on the excellent results observed, meets the requirements of advanced proteomics. The extreme complexity shown by most profiles has also accelerated the development of novel separation techniques. A step forward in this field is indeed represented by multidimensional HPLC in which the “conventional” reverse phase is integrated with other orthogonal separation modes. Among various two-dimensional (2D) HPLC procedures, Strong cation exchange (SCX)-RP-LC is the most widely used for bottom-up-based proteome analysis. As judged by the high number of articles dealing with this approach (see [25,48,54,57,60,74,75,78]), the advantages of this system can be evaluated not only in terms of high efficiency and resolution, but also peak capacity and high throughput.

Although most proteomic studies described in this report aimed at comparing different states of a proteome rather than achieving absolute quantitative data, with the ability to rapidly identify large numbers of proteins, the emphasis has also shifted to their estimation. The question of which method would be most suitable for obtaining data useful for a better quantification of the proteome under investigation is a difficult and important topic for which many parameters must be considered. The articles presented in the above sections contain a good number of the existing methods for the comparison of protein abundance in different samples. Broadly speaking, among the most straightforward and simple processes to quantify information, MS/MS-based label-free analysis comes first. The main reason is that this method does not require time-consuming sample labeling. It only requires that sample preparation and data acquisition are reproducible, which is usually expected. Of course, as no ideal method exists, data accuracy is the major drawback of this approach. Nevertheless, a good number of the above studies aimed at quantifying proteins have also applied the iTRAQ label-based approach (see [25,26,27,28,29,48,73,75]) in which multiple samples can be labeled, mixed and then analyzed simultaneously via MS to avoid technical issues related to reproducibility that may be encountered with label-free approaches. The ability of iTRAQ tags to react with all the primary amines of peptides (thus labeling all peptides and providing information about their post-translational modifications) allowed for the design of studies aimed at: (i) discovering biomarkers; (ii) understanding disease mechanisms; or (iii) improving methods for early and sensitive diagnosis. Obviously, the difficulty of identifying and quantifying proteins when uniquely expressed in one sample type only (e.g., a protein expressed only in the diseased state) still represents a limit for this approach. Owing to its ability to quantify protein levels and to monitor numerous potential biomarkers simultaneously in a single analysis, single reaction monitoring (SRM)/MRM was another strategy frequently applied in these studies (see [45,62,64,72,76,81]). However, the limit of this approach is that, to monitor the appropriate precursor/product ion, the proteins of interest should be known beforehand.

Among the set of tools that can be applied to identify, characterize and quantify the components in biological systems, LTQ-Orbitrap was the instrument of choice for most proteomic studies described in this report (see [29,46,48,63,65,68,73,74,93,95]). It should be emphasized that this instrument, with its ability to deliver low parts per million mass accuracy and an extremely high resolution, all within a time scale compatible with nano-LC separations, is the only commercially available system featuring the orbitrap mass analyzer. Given that the dynamic range over which accurate measurements of mass can be made determines the true utility of accurate mass capability for real-life applications, then a very accurately measured mass of a precursor ion allows for the considerable reduction of false positive peptide identifications. The ongoing quest for eliminating false positive identifications (considered to be the worst scourge of large-scale shot-gun proteomics experiments) can be certainly helped by acquiring all data with high mass accuracy. In this respect, LTQ-Orbitrap is proving itself as a tool useful for providing a significant contribution to this field.

As shown in the articles discussed above, the vast amount of data generated by proteomics experiments was translated into systems biology by applying bioinformatics tools on both differential expression and global expression profiles. Integrating the proteomics data into systems biology language is obviously the approach for understanding the behavior of complex organisms at various levels. In the case of gel-based proteomics (and not only), the Gene Ontology database was used to categorize the identified proteins according to their molecular functions and biological processes (see [48,54,57,82,88,89]). Current proteomic studies benefit from using gene ontology (GO), although the major drawback of this annotation system is that it does not describe the multiple forms of a gene, such as alternative splicing, proteolytic cleavage and post-translational modification. Therefore, Gene Ontology cannot describe the functional stage of the gene products. In other cases, hierarchical clustering (which enables proteins to be grouped or classified blindly according to their expression profiles) was applied as a useful approach in understanding the interdependencies of proteins in the expression profile, molecular classification and protein signature discovery of diseases and the dynamic changes of protein expression (see [74,82,89]). A number of database search tools and algorithms implementing the estimation of the false discovery rate have been developed and are commercially available. Among these, SEQUEST is a commonly used search algorithm for mass spectra and the Protein Lynx Global server is a system that combines raw data processing of multiplexed LC-MS datasets with protein/peptide identification and label-free quantification.

In a few peculiar cases (see [47]), noncommercial software was used to find peptide signals in data files encoding peak information.

## 10. Other Techniques for Biomarker Discovery

While being a great technology, HPLC is not the best for protein separation, and if we want to have an “accurate diagnosis and prognosis of a disease”, complementary techniques should be available. Among these, capillary electrophoresis (CE), with its ability to separate rapidly and with high efficiency compounds present in extremely small volumes, appears to be a valuable analytical tool for the analysis of complex biological mixtures. For example, by applying capillary zone electrophoresis (CZE) in a capillary containing 0.01% hydroxypropyl methyl cellulose linear polymer sieving solutions at the inlet portion and next to the outlet of the capillary, Deng *et al.* [96] have produced patterns of proteins from normal, squamous cell lung carcinomas (SQCLC) and SCLC tissues. Marked differences were observed between normal and pathological tissues. The versatility of immunoaffinity capillary electrophoresis (IACE) as a tool for determining protein biomarkers in inflammatory processes was shown in an interesting review article by Guzman and Phillips [97]. They underlined how the use of antibodies as highly selective capture agents, combined with the high resolving power of CE, permits the simultaneous quantification and characterization of several protein biomarkers, including variants, isoforms, peptide fragments and post-transaltional modifications (PTMs). Particular attention was dedicated to detection of cytokines as predictive biomarkers of inflammation.

Moreover, with the introduction of different MS interfacing platforms (e.g., ESI, MALDI), the popularity of CE, as a proteomic tool, has progressively increased. The interfacing of CZE, capillary isoelectrofucusing (CIEF) and on-column transient capillary isotachophoresis (CITP) to MS resulted in a rapid increase of CE-MS use for the analysis of a wide variety of compounds, including amino acids, protein digests, protein mixtures, single cells and oligonucleotides. As illustrated in a series of review articles [98,99,100], CE-based (single- and multi-dimensional) separations, coupled with MS, have been applied for performing comprehensive proteomic analyses of different biological specimens. This resulted not only in the identification of proteins, but also in monitoring their quantitative changes in expression. Advances in CE-MS for clinical proteomic applications have been reviewed in a very recent article by Stalmach *et al.* [101]. Although only a few of these advances are still focused on respiratory diseases, their potential for delivering improvements in proteomic work is clearly evident.

A few years ago, Li *et al.* [102] also reported the development of a microfabricated CE-nano-electrospray/mass spectrometer that included capillary electrophoretic channels and an interface to a nano-electrospray ionization tip. The analytical potential of this device was demonstrated with the identification of proteins from *Neisseria meningitidis.*

Since then, microfabrication and microfluidics principles have been increasingly applied in proteomics for the development of cost-effective, high-throughput strategies. In particular, clinical chemistry is moving globally toward the use of miniaturized portable devices that can quantify hundreds of biomarkers for one or more diseases of interest and that can be used not only in a big laboratory of a major hospital, but also in a doctor’s office and by the patient itself, if necessary, in remote locations. At the present time, microfluidic-based point-of-care diagnostic devices are routinely being used for the determination of multiple biomarkers on a variety of samples, ranging from saliva, blood, cerebral spinal fluid and tears [103,104].

A list of advantages and disadvantages of chromatographic *vs.* capillary electrophoretic techniques is reported in Table 3.

**Table 3:** Advantages and disadvantages of chromatographic and capillary electrophoretic techniques in proteomics. CE, capillary electrophoresis; IACE, immunoaffinity CE.

Method	Advantages	Disadvantages
LC-MS	-High sensitivity -High specificity -High resolution	-Very expensive equipment and columns -Relatively high volumes of sample injected (µL) -Very high expertise needed
CE-MS	-Less expensive than LC-MS -Very small volumes of samples injected (nL) -Lower expertise needed compared to LC-MS -Basic and hydrophilic peptides with low molecular masses easier to be detected than in LC-MS -Fast separation	-Limited loading capacity -Unavailability of an integrated system as a marketed solution
IACE	-Enrichment and quantification of ultra-low abundance analytes in complex biological matrices -Combines high-resolving power of CE and on-line coupling of high selective antibody-capture agents. -Very fast separation -Separation and quantification of intact substances and their respective modified counterparts. -Potential for measuring single-cell components -Miniaturization -Low cost	Apparently not one of the disadvantages indicated for other techniques is observed in this capillary electrophoretic approach.

## 11. Concluding Remarks and Future Perspectives

One school of thought suggests that, by creating complex “proteomic fingerprints” of healthy and diseased states (and the transitions thereof), one may recognize perturbations from the healthy state phenotype before the manifestation of the disease state. The huge amount of experimental data generated by the “furious activity” described in this report demonstrates that progression from health to disease is always accompanied by complex changes in protein expression in both the circulation and affected tissues. Showing the merits of MS techniques in producing qualitative and quantitative information on the protein patterns of a variety of human tissues/fluids, the above paragraphs provide a picture of respiratory proteomics to date. Taken together, the results documented here demonstrate that, in addition to proteomic profiles relevant for gaining insights into molecular events correlated with lung carcinogenesis, also the identification of metastasis-associated proteins or of altered proteins, whose implication as potential lung cancer biomarkers could be elaborated, have been successfully performed. Methods for a precise calculation of protein concentration and for the determination of the extent of modification, including, among others, phosphorylation, glycosylation and ubiquitination (all adducts that significantly contribute to protein function), have also been developed. Undoubtedly, the striking improvement in technologies related to accuracy, when coupled to quantitative approaches, has a great impact on the quality of the results. However, by reviewing these reports, the question also arises of whether sophisticated technologies (such as the cited iTRAQ labeling) may actually be more fruitful, in terms of candidate protein marker identification, than “conventional” procedures. In light of the versatility and high degree of reproducibility shown by this strategy, a positive answer is perhaps not surprising, at least for two reasons. First, the multiplexing tagging chemistry of iTRAQ allows, with minimal sample consumption, the simultaneous work-up of four to eight samples; second, the increased number of peptides identified and quantified the results with a higher accuracy, which translates into improved alignment and quantification across spectra. 

On the other hand, the high level of reproducibility is suitable for analyte identification and metabolic network reconstruction knowledge about the functions of many proteins. In addition, aside from the interest in deciphering the function of individual proteins, the set of data produced by these methods may represent the starting point for studying large-scale interactions that serve to discover general important properties for interaction participation. The finding that highly interactive proteins often are well conserved and/or essential or that homologous proteins, and, in particular, proteins with domains from the same family, tend to interact more frequently than others is likely to improve the knowledge of their intrinsic properties. Thus, the understanding of the role these proteins play in the pathogenesis of respiratory disease, while opening the door to much more powerful protein diagnostics, reinforces the linkage between basic medical research and clinical laboratory medicine. Addressing these concerns is obviously a top priority for the field, the ultimate goal of researchers being to understand the biology of disease and to translate this knowledge into the clinic. There is no doubt that this branch of respiratory proteomics will have substantial improvement in the future. Recent technical advances in the field of samples fixed with formalin followed by paraffin embedding demonstrate that a global proteomic study could be performed also on these specimen. FFPE is the most common procedure for long-term preservation of clinical samples. In this respect, the huge amount of samples present in hospital archives may become a real treasure for retrospective proteomic analysis, to elucidate pathological pathways or to retrieve disease-associated biomarkers. These studies may be of vital importance for biomarker discovery, particularly since the large banks of historical tissues could be potentially very useful for validation studies. Unfortunately, the extensive formaldehyde-induced protein/DNA/RNA crosslinking is a barrier for many analytical platforms and, although new approaches for unlocking the proteome of these tissues are being developed, successful removal of the crosslinks is still a challenge. To obtain detailed information about the pitfalls/quality of different FFPE proteomic platforms and of the analytical strategies used to unravel the FFPE proteome, the reader is directed to a very interesting recent review in this area [105].

The good number of proteins identified and quantified from the proteomic analysis of xenograft tumors indicates that these recent methodological advancements have made possible the realization of very efficient and versatile proteomic platforms.

With the rapid pace of technological improvements, two other areas of proteomics are attracting increasing attention. One is the discovery of biomarkers in human populations, and the other is imaging mass spectrometry (IMS), *i.e*., obtaining the MS spectrum from thin tissue sections to produce molecular weight-encoded “images” of the distribution of constituent biomolecules. Although both approaches are still in early development and so far have never been applied to respiratory diseases, their “explosion” as new strategies for investigating these lung disorders may have a large impact in the coming years.

In conclusion, the constant production of excellent articles in this field [106,107,108,109] confirms that respiratory proteomics may become a methodological tool that is decisive for the identification/ characterization of disease-associated proteins. Currently as a positive consequence of the enhancement of the depth and breadth of proteome coverage, proteomic signatures specific for different lung diseases have begun to emerge. 

However, while being encouraging, these findings still need extensive validation, and novel platforms will probably be developed to demonstrate their clinical utility.

## Supplementary Materials

Supplementary File 1
