# Supplementary material for: Respiratory Proteomics Today: Are Technological Advances for the Identification of Biomarker Signatures Catching up with Their Promise? A Critical Review of the Literature in the Decade 2004–2013
Source: Proteomes. 2014 Jan 22;2(1):18–52. doi: 10.3390/proteomes2010018 (PMC5302730; doi:10.3390/proteomes2010018)
Supplement: Supplementary File 1 [file proteomes-02-00018-s001.pdf]

Clarification: Viglio, S. *et al*. Respiratory Proteomics Today: Are Technological Advances for the Identification of Biomarker Signatures Catching up with Their Promise? A Critical Review of the Literature in the Decade 2004–2013

We wish to note that some phrases contained in our published paper [1] were taken from a review report without the appropriate attribution in the first published version. These were, on page 36: “It is often the case that these—low hanging fruit molecules that turn up time and time again in biomarker studies are of a high abundance, e.g., inflammatory proteins, which are symptomatic of aggressive cancers and advanced disease, rather than having etiologic roles in these disorders. These proteins are often multifunctional, making them poor targets for new therapies” and on page 44: “These studies may be of vital importance for biomarker discovery, particularly since the large banks of historical tissues could be potentially very useful for validation studies”. As this was discovered and corrected before the issue release, the original document has been updated.

We would like to thank the reviewers for their efforts to improve this paper, and apologize for inconvenience to the reviewers and readers.

Reference

1. Viglio, S.; Stolk, J.; Iadarola, P.; Giuliano, S.; Luisetti, M.; Salvini, R.; Fumagalli, M.; Bardoni, A. Respiratory Proteomics Today: Are Technological Advances for the Identification of Biomarker Signatures Catching up with Their Promise? A Critical Review of the Literature in the Decade 2004–2013. *Proteomes* **2014**, *2*, 18–52.

© 2014 by the authors; licensee MDPI, Basel, Switzerland. This article is an open access article distributed under the terms and conditions of the Creative Commons Attribution license (http://creativecommons.org/licenses/by/3.0/).
